# Phenotypic plasticity in response to temperature fluctuations is genetically variable, and relates to climatic variability of origin, in *Arabidopsis thaliana*

**DOI:** 10.1093/aobpla/ply043

**Published:** 2018-07-16

**Authors:** J F Scheepens, Ying Deng, Oliver Bossdorf

**Affiliations:** Plant Evolutionary Ecology, Institute of Evolution and Ecology, University of Tübingen, Auf der Morgenstelle, Tübingen, Germany

**Keywords:** Adaptation, climatic variability, genotype, heat stress, intraspecific variation, phenotypic plasticity

## Abstract

Under current climate change, increasing mean temperatures are not only causing hotter summers, but temperature variability is increasing as well. Phenotypic plasticity can help plants to overcome negative effects of temperature variability and allow them to rapidly adjust traits to adverse conditions. Moreover, genetic variation in such plasticity could provide potential for adaptive evolution in response to changing climate variability. Here, we conducted an experiment with 11 *Arabidopsis thaliana* genotypes to investigate intraspecific variation in plant responses to two aspects of variable temperature stress: timing and frequency. We found that the timing but not frequency of temperature stress affected the phenology, growth, reproduction and allocation strategy of plants, and that genotypes differed substantially in their responses. Moreover, trait plasticity was positively related to precipitation variability of origin, suggesting an adaptive role of plasticity. Our results indicate that the developmental stage of a plant during heat stress is a key determinant of its response, and that plasticity to temperature variability is an evolving and possibly adaptive trait in natural populations of *A. thaliana*. More generally, our study demonstrates the usefulness of studying plant responses to climatic variability *per se*, given that climatic variability is predicted to increase in the future.

## Teaser

Climate change not only means warmer temperatures but also stronger temperature fluctuations. However, we do not know yet whether plants can adapt to these. We carried out an experiment in which *Arabidopsis thaliana* plants from 11 origins were grown under different fluctuating temperature stress conditions. We found that plant origins differed in their responses to changes in stress timing, and that responses were stronger for plants that came from regions with more variable climates. Our results suggest that plants may have adapted to climate fluctuations in the past, and that they have the potential to do this again in the future.

## Introduction

Global climate change is significantly affecting plants and animals across the globe (Parmesan and Yohe 2003; Root *et al.* 2003; Menzel *et al.* 2006; Reyer *et al.* 2013). Under current climate change, increasing mean temperatures are not only causing hotter summers, but temperature variability is increasing as well (Schär *et al.* 2004; Fischer and Schär 2009). This increase in variability can take place at different temporal scales, e.g. diurnally, intra-seasonally or inter-annually. As a consequence, temperature extremes are currently occurring more regularly and are predicted to increase even more in frequency in the future (Fischer and Schär 2009; Barriopedro *et al.* 2011).

While plant and community responses to changing mean temperature and precipitation have already been well investigated (Walther *et al.* 2002; Wu *et al.* 2011), much less work has been done so far on plant responses to changes in climatic variability (Jentsch *et al.* 2007; Reyer *et al.* 2013). Some previous studies indicate that increasing climatic variability *per se* may have strong repercussions for plant and community performance (Knapp *et al.* 2002; Chesson *et al.* 2004; Sher *et al.* 2004) and that climatic variability may sometimes affect population dynamics and community functioning even more strongly than climatic means (Fay *et al.* 2000; Sher *et al.* 2004). Moreover, as plant populations are often adapted to their climates of origin (Manel *et al.* 2010; Fournier-Level *et al.* 2011; Hancock *et al.* 2011; Ågren and Schemske 2012; Toräng *et al.* 2014), and this may include not only adaptation to the means of temperature and precipitation (Manel *et al.* 2010) but also to their temporal variability (Pratt and Mooney 2013; Manzano-Piedras *et al.* 2014), climate change may disrupt such adaptations.

If temperature fluctuations and high temperature stress have negative effects on plant growth (Kotak *et al.* 2007), then the current and predicted increase in the frequency of temperature extremes will impact plant fitness and survival, with potential repercussions on population persistence (Jump and Peñuelas 2005; but see Cahill *et al.* 2012). However, populations may differ genetically in their tolerance to temperature fluctuations, and such variation may reflect past selection by the climatic variability of the site (Gianoli and González-Teuber 2005; Pratt and Mooney 2013; Lázaro-Nogal *et al.* 2015). For instance, a study on a semi-arid Chilean shrub, *Senna candolleana*, showed that populations from climatically more variable sites showed greater adaptive plasticity to water availability and may therefore be able to cope better with future increasing climatic variability despite being exposed to higher levels of stress (Lázaro-Nogal *et al.* 2015). Such intraspecific variation in responses to climatic variability may prove crucial for future adaptation to changing climatic variability, and it suggests that populations in climatically variable environments may suffer less from increasing variability than populations from more stable climatic conditions. A formal proof of adaptive plasticity in response to climatic variability would require to demonstrate positive relationships between the degree of plasticity across different climates and the mean fitness across these environments (Sultan 2000; Relyea 2002; Van Kleunen and Fischer 2005).

Climatic variability is a broad term, and a change in variability may have different aspects. For instance, for discrete environmental events, variability may change through changes in the events’ duration, frequency, timing and/or intensity (Shea *et al.* 2004). Each of these aspects may have different effects on the organisms, and experiments allow us to control and study them separately. Whatever the exact experimental design is, an important notion is that experiments investigating the effects of changes in climatic variability should avoid confounding changes in the variability of a climate variable with changes in its mean by keeping the overall mean of an experimentally altered climate variable, e.g. the average temperature or precipitation sum, constant across the experiment (Parepa *et al.* 2013), or by combining changes in means with changes in variability in a multifactorial experimental design. So far, such experiments are still rare.

Here, we conducted an experiment in which we investigated intraspecific variation in plant responses to two aspects of variable temperature stress: timing and frequency. We used *Arabidopsis thaliana* as a model system, because natural genotypes from various geographic locations with contrasting climates are readily available from seed stock centres and these exhibit large genetic variation (1001 Genomes Consortium 2016). In general, genotype–environment interactions and their genetic basis have already been well studied in *A. thaliana*. For instance, flowering time responses across 473 natural genotypes grown under two contrasting temperature and light environments mimicking Spanish and Swedish climates suggest adaptation (Li *et al.* 2010), and this result has been corroborated in a field study in Italy and Sweden (Ǻgren and Schemske 2012). Vile and co-workers (2012) found variable responses to temperature and drought treatments in various traits among 10 natural genotypes. The production of heat shock proteins (HSPs) in response to heat stress was found to be variable among genotypes and related to heat-stress resistance as well as to heat-stress levels experienced under natural conditions (Tonsor *et al.* 2008). Thus, genotype by environment interactions are abound in *A. thaliana*, but virtually all studies investigated responses to changes in environmental means, whereas studies on genotype-specific responses to changes in environmental variability are so far lacking.

We used 11 *A. thaliana* genotypes from the species’ natural range, and exposed the plants to six different scenarios of temporally variable temperature stress while keeping the average temperature constant across all treatments. The overall aim of the study was to investigate how plants responded in terms of performance, phenology and architecture to changes in the timing versus frequency of temperature stress, and whether there was intraspecific variation in these responses that would indicate evolutionary potential for adapting to changing climatic variability. We also tested whether plasticity to temperature variability was adaptive, and whether it was related to the climate of origin of the 11 studied genotypes. Specifically, we asked the following four questions: (i) How does *A. thaliana* respond to changes in the timing versus frequency of temperature stress? (ii) Do genotypes differ in their responses to these changes? (iii) If yes, is the degree of plasticity to the different temperature stress treatments related to the fitness robustness of *A. thaliana* genotypes across environments? (iv) Is the tolerance of *A. thaliana* genotypes to temperature stress related to their climatic origin?

## Methods

### Experiment

To examine tolerance to temperature variability, and genetic variation therein, of *A. thaliana*, we performed a full-factorial experiment in which 11 *A. thaliana* genotypes were subjected to temperature stress at different times and frequencies. We initially selected 12 genotypes from the Versailles ‘core collections’ maximizing genetic diversity (McKhann *et al.* 2004) **[see****Supporting Information—Table S1****]**. Specifically, we worked with Blh-1, Bur-0, Ct-1, Ita-0, JEA, Oy-0 and Sha from the ‘core collection 8’, plus Can-0, Ge-0, Mt-0, N13 and St-0 from the ‘core collection 16’. All selected lines were of native origin and did not require vernalization to flower. During our experiment, all plants from the genotype Ita-1 (but none of the others) died of an unidentified fungal disease and were therefore removed from the experiment and subsequent analyses, leaving 11 genotypes.

We placed seeds from all genotypes on moist filter paper in Petri dishes and stratified them for 5 days at 4 °C in the dark. Thereafter, we sowed the seeds into 5 × 5 × 4.5 cm pots filled with a 45:45:10 mixture of potting soil, low-nutrient germination soil (Einheitserde, Sinntal-Altengronau, Germany) and sterilized sand. We initially planted two seeds of the same genotype in each pot, with 59 pots per genotype, and 708 pots in total. Prior to the start of the experimental treatments, we thinned down all plants to one seedling per pot.

For our experiment, we used two walk-in growth chambers that were identical except for their temperature settings. The ‘normal’ chamber was set to 20/15 °C at a 14/10 h day/night cycle, whereas the ‘stress’ chamber was set to 30/25 °C with the same light conditions. The day temperature of 30 °C experienced in the stress chamber is known to exert stress on *A. thaliana* (Whittle *et al.* 2009; Vile *et al.* 2012), and this was confirmed in our experiment where periods spent in the stress chamber often resulted in aborted flowers and fruits. Under day conditions, the light intensity in the growth chambers was ca. 230 μmol·m^−2^·s^−1^ of photosynthetically active radiation. Air moisture was kept within 40–60 %.

**Figure 1.**
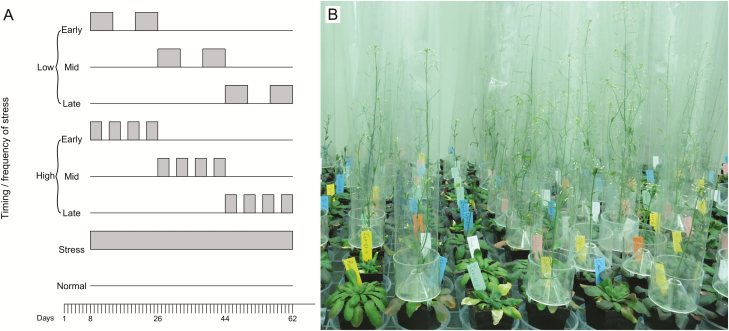
(A) Schematic of the six temperature fluctuation treatments—with three timings (early/mid/late) and two frequencies (low/high) of temperature stress—and two continuous control treatments at normal and stressful temperature. The grey blocks indicate the periods during which the plants experienced temperature stress. (B) Close-up of some of the experimental plants (photo: J.F.S.).

One set of control plants, with eight replicates per genotype, was placed in the normal chamber, while another set of control plants, with three replicates per genotype, was placed in the stress chamber for the whole duration of the experiment. The remaining 48 plants per genotype were all subjected to the same amount of 12 days of temperature stress, but with different temporal patterns of the stress periods, which were achieved by moving the plants from the normal chamber to the stress chamber at different times. Besides the two controls, there were six different stress treatments (Fig. 1): a factorial combination of three different timings of stress (early/intermediate/late) and two different frequencies (low/high), with eight replicates per genotype in each treatment. After a 1-week establishment phase for all plants in the normal chamber, the early-stress plants were moved to the stress chamber at day 8, and the intermediate- and late-stress plants at days 26 and 44, respectively. For each of these timing treatments, we imposed temperature stress at two different frequencies, either with two periods of 6 days of stress, and 6 days of recovery at normal conditions in between, or with four periods of 3 days of stress, and 3 days of recovery between each of these (Fig. 1). After the late-stress period, all plants except the control plants in the stress chamber remained in the normal chamber until they were harvested.

Throughout the experiment, we watered all plants regularly, so that water presumably never became a limiting factor. Every morning, we recorded the phenological state of each plant. The plants were classified as flowering when the first flower opened. At the end of the intermediate-stress period (day 43), we took leaf samples from a subset of the early- and intermediate-stress plants for subsequent molecular analyses (not reported here). Each plant was harvested 1 week after the first fruit ripened. We counted the numbers of fruits >2 mm as well as the numbers of basal and lateral shoots. We separated above-ground vegetative biomass (the rosette) from the reproductive biomass (inflorescences). The vegetative biomass was immediately dried for 72 h at 60 °C and weighed, whereas the inflorescences were first stored at room temperature for after-ripening and seed harvesting (for follow-up experiments) and then also dried and weighed.

### Data analysis

We analysed plant responses to temperature stress with regard to the following five response variables: (i) flowering time, (ii) plant architecture, i.e. the ratio of lateral to basal shoot number, with lower numbers indicating more ‘shrubby’ plants, (iii) above-ground biomass, (iv) reproductive allocation, i.e. the proportion of reproductive to total above-ground biomass and (v) fecundity, i.e. the number of fruits. To account for the biomass removal through leaf sampling from some early- and intermediate-stress plants, we included leaf sampling as a binary variable in all analyses.

First, we verified our experimental treatments, and whether the stress chamber conditions were indeed stressful for the plants, by analysing only the fecundity of the plants in the continuous normal versus continuous stress conditions. In this linear model, we also tested for genotypic differences in fecundity, and for the interaction between genotype and the continuous temperature treatments.

Next, we analysed the data from the six temperature fluctuation treatments with linear models that included leaf sampling, genotype, timing and frequency of stress as well as all possible two-way and three-way interactions between genotype, stress timing and stress frequency. To improve normality of the model residuals, flowering time was log-transformed and plant architecture was square root-transformed prior to the analyses.

To investigate whether increased trait plasticity is associated with higher robustness in terms of plant fitness, we used linear regressions that related a stan d ardized measure of fitness robustness of each genotype across environments to its trait plasticity across environments. To calculate fitness robustness, we divided the mean fitness across the six treatments by the maximum fitness achieved in one of the six treatments. This index renders the genotypes comparable among each other. The degree of trait plasticity was quantified using the coefficient of variation (CV) based on the mean trait values in the six treatments (Valladares *et al.* 2006).

Finally, we tested whether the observed degree of trait plasticity of different genotypes was related to their climate of origin. We used temperature and precipitation data from WorldClim (Hijmans *et al.* 2005) and calculated, for each genotype, the mean and SD of temperature as well as the mean and CV of precipitation for the months of the growing season. For each genotype’s location of origin, the growing season was determined based on threshold monthly values of minimum (>5 °C) and maximum temperature (<30 °C), precipitation (>20 mm per month) and water deficit (>−50 mm per month), with water deficit calculated as precipitation minus evapotranspiration, and evapotranspiration calculated according to Droogers and Allen (2002). In case all four thresholds were met for a given month, this month was included in the growing season and the calculation of climate variables. The growing season was, however, fixed to a length of 4 months starting with the earliest suitable month **[see****Supporting Information—Table S1****]**.

All analyses were performed in the software R v 3.4.3 (R Core Team 2017).

## Results

Plants continuously growing in the stress chamber had a significantly lower average fecundity (159.6 ± 24.0) than the plants continuously growing in the normal chamber (169.0 ± 12.8; ANOVA, *F*_1, 85_ = 5.48, *P* = 0.022), confirming that the higher temperatures in our experiment indeed exerted significant stress and decreased plant fitness. However, the overall effect of temperature stress differed among genotypes (*F*_8, 85_ = 2.22, *P* = 0.034), with some genotypes showing strong negative responses and others showing only weak or no responses, and one genotype even showing a positive response **[see****Supporting Information—Fig. S1****]**.

**Figure 2.**
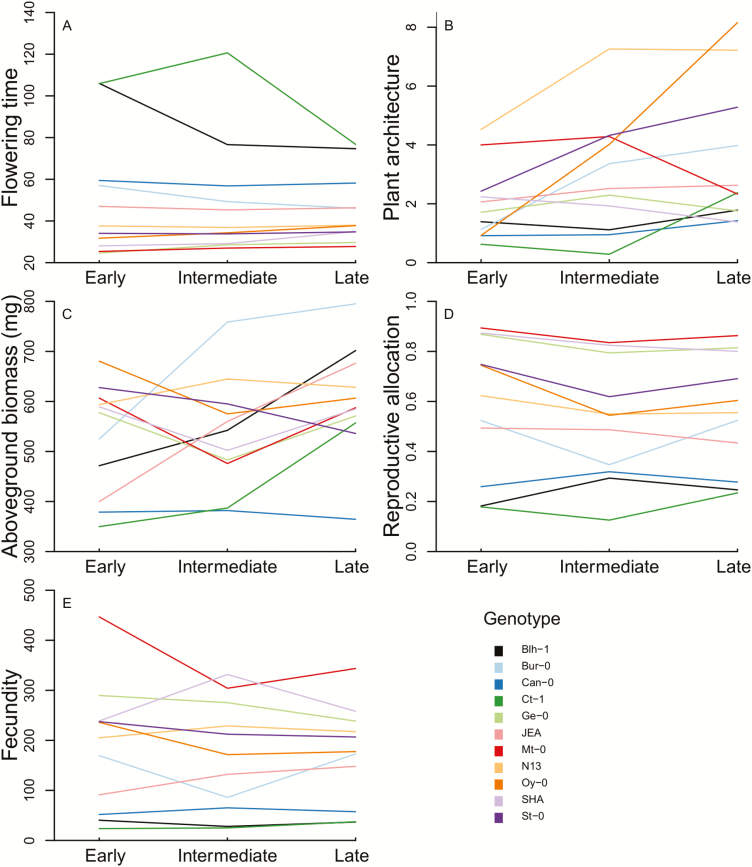
Response of 11 *Arabidopsis thaliana* genotypes to three different timings of temperature stress in five traits: (A) flowering time; (B) plant architecture; (C) above-ground biomass; (D) reproductive allocation; (E) fecundity.

The analyses of the plants in the six variable stress treatments showed that overall, timing but not frequency of temperature stress affected performance, phenology and architecture of the plants (main effect of stress timing and its interaction with genotype; Table 1). Across all genotypes, the timing of stress significantly affected fecundity as well as reproductive allocation and plant architecture, with the highest average fecundity and the lowest ratio of lateral to basal shoots in early-stressed plants, and lowest reproductive allocation at intermediate stress timing (Fig. 2). However, some individual genotypes deviated from these general trends. We also found significant interactions between stress frequency and timing in fecundity and reproduc tive allocation: higher frequency had a positive effect on both of these traits if the stress occurred early, but it had no or even the opposite effect if the stress occurred at later **[see****Supporting Information—Fig. S2****]**. There were strong genotype effects in all of the measured traits, and the effects of stress timing were also generally strongly genotype-dependent (Table 1; Fig. 2). Finally, there was a three-way interaction among stress timing and frequency, and genotype identity for reproductive allocation (Table 1), which therefore modulates the two-way interaction of stress timing and frequency **[see****Supporting Information—Fig. S3****]**. Results hardly differed when plants which leaves were sampled for subsequent molecular analyses were removed from the analysis **[see****Supporting Information—Table S2****]**.

**Table 1.** Results of linear models testing the phenotypic responses of 11 *Arabidopsis thaliana* genotypes to different timings (early/mid/late) and frequencies (low/high) of temperature stress. Shown are *F*-ratios and *P*-values, the latter highlighted in bold when below 0.05.

		Flowering time		Plant architecture		Above-ground biomass		Reproductive allocation		Fecundity	
	d.f.	*F*	*P*	*F*	*P*	*F*	*P*	*F*	*P*	*F*	*P*
Leaf sampling	1	0.39	0.533	9.70	**0.002**	69.05	**<0.001**	2.27	0.133	1.47	0.226
Stress timing (T)	2	0.22	0.805	20.31	**<0.001**	2.67	0.071	14.74	**<0.001**	3.23	**0.041**
Stress frequency (F)	1	1.46	0.227	0.01	0.931	1.04	0.308	0.83	0.364	0.68	0.409
T × F	2	1.67	0.189	0.37	0.692	0.85	0.428	6.74	**0.001**	5.66	**0.004**
Genotype (G)	10	356.23	**<0.001**	45.19	**<0.001**	23.61	**<0.001**	297.90	**<0.001**	131.11	**<0.001**
G × T	20	7.97	**<0.001**	6.53	**<0.001**	6.63	**<0.001**	4.73	**<0.001**	4.99	**<0.001**
G × F	10	1.59	0.107	1.54	0.123	0.65	0.769	0.82	0.609	0.73	0.694
G × T × F	20	0.78	0.743	1.18	0.265	1.34	0.148	2.47	**<0.001**	0.87	0.621
Residuals	447–454										

**Figure 3.**
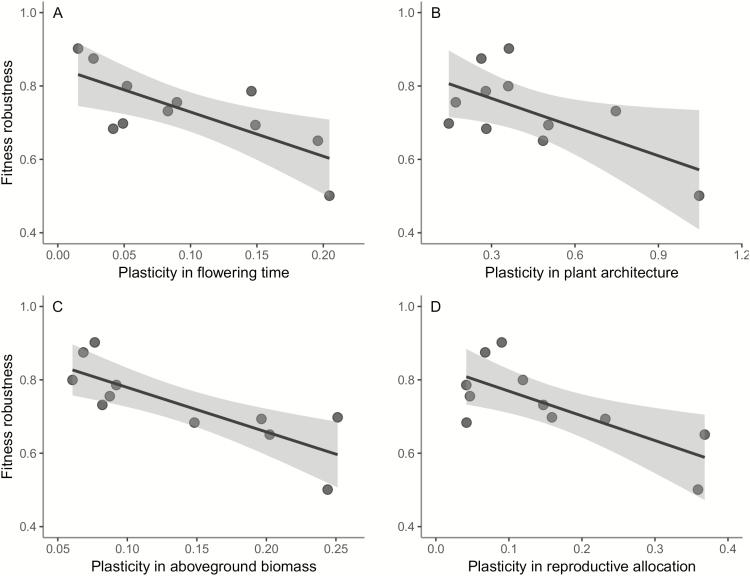
Relationships between fitness robustness across environments and trait plasticity—(A) flowering time; (B) plant architecture; (C) above-ground biomass; (D) reproductive allocation—for 11 genotypes of *Arabidopsis thaliana*.

**Figure 4.**
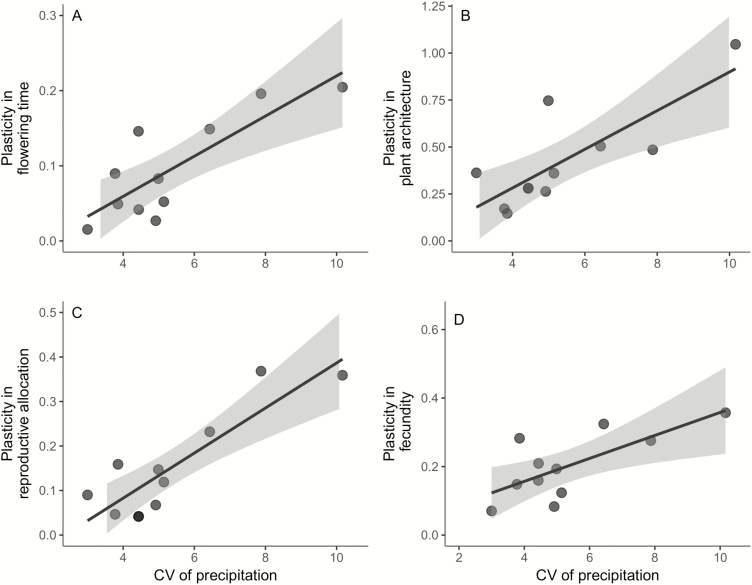
Relationships between trait plasticity—(A) flowering time; (B) plant architecture; (C) reproductive allocation; (D) fecundity—and precipitation variability of origin for 11 genotypes of *Arabidopsis thaliana*.

Fitness robustness was negatively related to plasticity in flowering time (*F*_1, 9_ = 10.68, *P* = 0.010; Fig. 3A), plant architecture (*F*_1, 9_ = 5.97, *P* = 0.037; Fig. 3B), above-ground biomass (*F*_1, 9_ = 16.71, *P* = 0.003; Fig. 3C), reproductive allocation (*F*_1, 9_ = 10.21, *P* = 0.011; Fig. 3D), and. When relating trait plasticity to the climates of genotype origin, we found that for four out of five traits (i.e. all except above-ground biomass), trait plasticities were positively related to the precipitation variability of origin (Table 2; Fig. 4). Except for one significant positive relationship of plant architecture with mean precipitation of origin (*R*^2^_adj_ = 0.32; *F*_1, 9_ = 5.70, *P* = 0.041; Table 2), trait plasticity was unrelated to any of the other climate variables.

**Table 2.** Results of linear regressions testing for relationships between the climates of origin of 11 *Arabidopsis thaliana* genotypes, and their trait plasticities in response to fluctuating temperature stress. Shown are adjusted *R*^2^-values, *F*-ratios and *P*-values, the latter highlighted in bold when below 0.05.

	Plasticity														
	Flowering time			Plant architecture			Above-ground biomass			Reproductive allocation			Fecundity		
	*R* ^2^_adj_	*F*	*P*	*R* ^2^_adj_	*F*	*P*	*R* ^2^_adj_	*F*	*P*	*R* ^2^_adj_	*F*	*P*	*R* ^2^_adj_	*F*	*P*
Mean temperature	−0.10	0.06	0.806	−0.11	0.05	0.831	0.0	0.96	0.353	0.23	4.03	0.076	−0.08	0.23	0.645
SD of temperature	−0.11	0.02	0.884	0.02	1.16	0.309	−0.11	0.02	0.895	0.02	1.21	0.300	−0.08	0.22	0.647
Mean precipitation	0.14	2.64	0.139	0.32	5.70	**0.041**	−0.11	0.02	0.887	−0.03	0.71	0.423	−0.03	0.70	0.424
CV of precipitation	0.64	18.57	**0.002**	0.59	15.64	**0.003**	0.27	4.65	0.059	0.74	29.82	**<0.001**	0.47	9.69	**0.012**

## Discussion

The goal of our study was to better understand how plants respond to changes in the temporal variability of the environment, and the extent and structure of intraspecific variation in this respect. We found that the timing of temperature stress strongly affected the growth and reproduction, resource allocation, phenology and architecture of *A. thaliana*, but the frequency of temperature stress did not. There was large variation in plasticity to stress timing among the 11 tested *A. thaliana* genotypes, and their degree of plasticity in this experiment was negatively related to fitness robustness, but positively related to the precipitation variability of their origins. Below, we discuss each of these results, and their implications, in detail.

### Timing, not frequency, of temperature stress matters

*Arabidopsis thaliana* responded to different timing but not to frequency of temperature stress. It is likely that the observed effects of stress timing were related to plant development. The developmental stage is important for a plant’s response to heat stress (Wollenweber *et al.* 2003; Hedhly *et al.* 2009). For instance, Wollenweber and co-workers (2003) found that heat stress did not affect wheat yield when applied during the vegetative stage but caused strong yield declines when applied during flowering. Similarly, we found that plants that were flowering during a stress treatment tended to abort these flowers (personal observation), leading to reduced fitness. Nine out of 11 genotypes started flowering during days 24–60, i.e. largely during the period when the intermediate and late stress treatments were applied to some of the plants, and the remaining two genotypes started flowering after all treatments were done; virtually no flowering took place during early stress. This may explain the overall reduction in fecundity under intermediate and late when compared to early temperature stress. Nevertheless, results for fecundity, above-ground biomass, reproductive investment and plant architecture did not change when we added flowering time as a covariate in the models **[see****Supporting Information—Table S3****]**. Perhaps other developmental stages, such as flowering duration, are more important determinants of the outcome of stress timing on plant traits.

The absence of an effect of stress frequency may be explained by an acquired thermotolerance, where after initial exposure to temperature stress, thermotolerance is retained, or decaying only slowly over time (Burke *et al.* 2000; Charng *et al.* 2006). This could explain why a different number of subsequent exposures to stress does not lead to a different response. The mechanism underlying acquired thermotolerance could be HSPs. It is well known that plants produce HSPs after exposure to high temperatures (Kotak *et al.* 2007), and that HSPs play a central role in heat-stress resistance through their function as molecular chaperones, i.e. they stabilize other proteins and thereby safeguard their functioning (Sørensen *et al.* 2003; Kotak *et al.* 2007).

### Genotypic variability

All traits showed substantial genotypic variation in their responses to timing of stress. As explained above, plants often respond differently to environmental stimuli depending on the developmental stage they are in (Hedhly *et al.* 2009). Since the genotypes in this experiment differed in their developmental rates, as evidenced by the variation in flowering time, this likely explains part of the genotypic variation in the response to timing of temperature stress observed in our experiment. Nevertheless, not all genotypic variation can be explained by the phenological stage during stress treatments. For instance, three genotypes (Bur-0, Can-0 and JEA) which started flowering during days 44–62 (i.e. the period of late stress) did not show decreased fitness when they were subjected to heat stress during flowering; JEA even increased fitness and Bur-0 showed a fitness decrease when it received stress at the intermediate timing, before flowering. Contrasting responses in terms of fitness were also observed in the six genotypes which all flowered primarily during the intermediate stress timing, with two genotypes increasing (N13, Sha), three decreasing (Mt-0, Oy-0, St-0) and one hardly responding (Ge-0) to intermediate as compared to early stress. In line with this genotypic variation, adding flowering time as a covariate in the models of the other four traits did not lead to loss of the genotype by stress timing interaction and therefore could not explain the results **[see****Supporting Information—Table S3****]**. Thus, genotypes vary in the sensitivity of their reproductive phase to heat stress, and other developmental stages than flowering can be sensitive to heat stress, too. Such variation could, for instance, be related to genotypic differences in HSP genes and activity (Sørensen *et al.* 2003). Genotypes from more southern latitudes are likely to be naturally exposed, and therefore adapted, to the applied temperature stress treatment in contrast to genotypes from more northern latitudes (Li *et al.* 2010; Ǻgren and Schemske 2012). However, adding latitude as a covariate in the models did not lead to loss of the genotype by stress timing interaction **[see****Supporting Information—Table S4****]**, so genotypic clines with latitude therefore do not fully explain these genotypic responses.

Whether mediated through developmental stage or through other mechanisms, our results clearly indicate that there is substantial genotypic variation within *A. thaliana* in the response to timing of heat stress. This variation is heritable and therefore constitutes evidence for evolutionary potential which could in principle lead to adaptation to different environments with contrasting temporal patterns of heat stress. However, we should note that the genotypes used in this study originated from diverse geographic locations, so the observed variation likely overestimates the levels of variation within natural populations (where evolution by natural selection takes place). Nevertheless, natural populations of *A. thaliana* are usually not genetically uniform (Bomblies *et al.* 2009; Montesinos *et al.* 2009), offering potential for adaptation. Moreover, seed dispersal may to some degree allow adaptive genotypes to track favourable climates. Overall, given projected climate change, it is likely that the timing of heat stress, rather than its frequency, will exert selection pressures on natural populations and result in rapid evolution of their phenotypic plasticity.

### Relationship between fitness robustness and plasticity

The negative relationship between fitness robustness and the width of plasticity across the treatments indicates that more plastic genotypes have less stable fitness across environments. In other words, genotypes with stronger trait plasticity suffered on average greater reduction in fitness across environments compared to their optimum (in this experiment), whereas genotypes with weaker plasticity had more robust fitness across environments. It may be that these plant responses to the variable temperature stress treatments are merely passive (e.g. reduced growth under abiotic stress) and go together with a fitness loss. Alternatively, plasticity could be beneficial but costly (Ghalambor *et al.* 2007). Indeed, HSPs are resource demanding and are toxic at high concentrations (Hoffmann 1995; Feder and Hofmann 1999). Ghalambor and co-workers (2007) described that strong fitness loss may result when an otherwise adaptive response becomes maladaptive when it falls outside the usually experienced range of environments. However, the two temperature treatments applied in this experiment do not constitute extreme environments for most if not all of the genotypes, rendering this explanation unlikely.

Alternatively, the results may reflect an advantage of phenotypic robustness in the face of the experimentally applied environmental variability, whereas phenotypic plastic responses, whether passive or active, cause fitness losses, at least in this experiment. This may relate to the temporal grain of environmental changes being too fast for plastic responses to be adaptive (Alpert and Simms 2002). In other words, the short periods under temperature stress in this experiment may penalize more plastic genotypes since their responses may be too slow to track the temporal environmental changes the plants were subjected to. Slow or non-responding genotypes may then achieve a higher fitness across the environments and thus be better adapted to such rapid temporal fluctuations in the environment. The question remains, then, whether 3 or 6 days of consecutive temperature stress as applied in this study is at odds with heat stress as experienced under natural conditions.

Stronger fitness homeostasis in phenotypically more robust genotypes could also indicate that these genotypes are able to achieve strong plastic responses at the physiological level (Thompson 1991). This seems to be at odds with the positive relationships between plasticity and precipitation variability of origin, which suggest adaptive plasticity of the observed traits.

### Relationship between plasticity and climate of origin

We observed that genotypes originating from environments with stronger precipitation variability showed stronger plasticity in most analysed traits. Such relationships fit the classical expectation that more heterogeneous environments should select for more plastic genotypes (Alpert and Simms 2002). It makes theoretical sense that plants in more temporally variable environments are able to adjust reproductive allocation, flowering time and plant architecture more flexibly (Alpert and Simms 2002). For instance, a drought spell may trigger an escape strategy in annuals (Franks 2011), advancing flowering to secure reproduction despite a strong fitness reduction compared to an otherwise more benign envir onment. The experience of drought may also translate into an altered reproductive allocation and an altered plant architecture (Williams and Black 1994). A key role for variability in water availability was likewise found in studies on other plant species (Gianoli and González-Teuber 2005; Pratt and Mooney 2013; Lázaro-Nogal *et al.* 2015). Since in the current experiment, plants were well watered, their responses should therefore not be directly related to drought but rather to temperature stress. Nevertheless, mechanisms and genetic pathways responding to drought and heat stress show considerable overlap in *A. thaliana* (Rizhsky *et al.* 2004). Heat stress in our experiment could therefore have partially triggered responses that in nature co-occur during drought stress, which may have driven evolution of plasticity. This could explain why the trait plasticities correlated with precipitation variability and not temperature variability of origin: precipitation variability may have been the selective agent for plastic responses while at the same time such responses can be triggered by temperature variability, even though temperature variability itself did not cause evolution of plasticity. An alternative explanation could be that temperature variability of origin, as derived from monthly mean values, does not capture temperature fluctuations relevant for adaptation to temperature variability. However, correlations between trait variability and mean diurnal temperature were never significant (*P* > 0.238; results not shown). Finally, it should be noted that our limited sample size of 11 genotypes may have constrained the discovery of further plasticity–environment relationships.

## Conclusions

Our study shows that phenotypic plasticity in fitness, growth, resource allocation, phenology and architecture in response to temperature variability—in particular to the timing of temperature stress—is variable among *A. thaliana* genotypes and therefore holds evolutionary potential. The observed cross-genotype relationships between responses to variability and climatic variability of origin suggest that evolution has shaped this type of phenotypic plasticity in the past, and that the observed responses possibly reflect adaptive natural variation. Moreover, variability in plasticity might allow natural populations to continue to evolve plasticity under increasingly variable climates in the future. More generally, our study demonstrates the usefulness of studying plant responses not only to changes in mean climate but also to climatic variability *per se*, which is an important finding, given that climatic variability is predicted to increase in the future.

## Data

The raw data of this publication can be found in the **Supporting Information—Table S5**.

## Sources of Funding

This research was financially supported through a research fellowship of the Alexander von Humboldt Foundation to J.F.S.

## Contributions by the Authors

J.F.S. and O.B. designed the experiment. J.F.S. and Y.D. performed the experiment. J.F.S. analysed the data and drafted the manuscript. Y.D. and O.B. contributed to the final version of the manuscript.

## Conflict of Interest

None declared.

## Supporting Information

The following additional information is available in the online version of this article—

**Table S1.** *Arabidopsis thaliana* genotypes used in our experiment, with their IDs in the 1001 Genomes project (ID-1; 1001genomes.org) and the NASC (ID-2; www.arabidopsis.info) and Versailles (ID-3; publiclines.versailles.inra.fr) stock centres.

**Table S2.** Results of linear models testing the phenotypic responses of 11 *Arabidopsis thaliana* genotypes to different timings (early/mid/late) and frequencies (low/high) of temperature stress on plants which were not sampled for leaves for use in follow-up experiments (see main text).

**Table S3.** Results of linear models testing the phenotypic responses of 11 *Arabidopsis thaliana* genotypes to different timings (early/mid/late) and frequencies (low/high) of temperature stress including flowering time as a covariate.

**Table S4.** Results of linear models testing the phenotypic responses of 11 *Arabidopsis thaliana* genotypes to different timings (early/mid/late) and frequencies (low/high) of temperature stress including latitude as a random effect.

**Table S5.** Excel file containing the raw data from this experiment.

**Figure S1.** Fecundity of 11 *Arabidopsis thaliana* genotypes under continuous normal conditions (*n* = 8) and continuous stress conditions (*n* = 3).

**Figure S2.** Mean responses of (A) reproductive allocation and (B) fecundity to stress timing and frequency across 11 *Arabidopsis thaliana* genotypes.

**Figure S3.** Response of 11 *Arabidopsis thaliana* genotypes to three different timings and two different frequencies of temperature stress in reproductive allocation.

Supplement Material

Table S5
