# Supplementary material for: Phenotypic plasticity in response to temperature fluctuations is genetically variable, and relates to climatic variability of origin, in Arabidopsis thaliana
Source: AoB Plants. 2018 Jul 16;10(4):ply043. doi: 10.1093/aobpla/ply043 (PMC6084592; doi:10.1093/aobpla/ply043)

**SUPPORTING INFORMATION**

# Phenotypic plasticity in response to temperature fluctuations is genetically variable, and relates to climatic variability of origin, in *Arabidopsis thaliana*

**Table S1.** *Arabidopsis thaliana* genotypes used in our experiment, with their IDs in the 1001 Genomes project (ID-1; 1001genomes.org) and the NASC (ID-2; www.arabidopsis.info) and Versailles (ID-3; publiclines.versailles.inra.fr) stock centers. The growing season delimits the months of the year included in the calculation of climate means and variabilities.

| Name | ID-1 | ID-2 | ID-3 | Country | Latitude | Longitude | Growing season |
| --- | --- | --- | --- | --- | --- | --- | --- |
| Blh-1 | - | N1030 | 180AV | Czech Republic | 48.30 | 19.85 | 5-8 |
| Bur-0 | 7058 | N1028 | 172AV | Ireland | 54.1 | -6.2 | 5-8 |
| Can-0 | 7063 | N1064 | 163AV | Spain | 29.21 | -13.48 | 11-2 |
| Ct-1 | 7067 | N1094 | 162AV | Italy | 37.51 | 15.09 | 12-3 |
| Ge-0 | 8297 | N1186 | 101AV | Switzerland | 46.21 | 6.14 | 6-9 |
| Ita-0 | - | N1244 | 157AV | Morocco | 34.09 | -4.20 | 11-2 |
| JEA | - | - | 25AV | France | 43.68 | 7.33 | 3-6 |
| Mt-0 | - | N1380 | 94AV | Libya | 32.34 | 22.46 | 11-2 |
| N13 | - | N22491 | 266AV | Russia | 61.36 | 34.15 | 6-9 |
| Oy-0 | 7288 | N1436 | 224AV | Norway | 60.39 | 6.19 | 5-8 |
| Sha | - | N929 | 236AV | Tajikistan | 38.59 | 68.79 | 2-5 |
| St-0 | 8387 | N1534 | 62AV | Sweden | 59.34 | 18.06 | 5-8 |

**Table S2.** Results of linear models testing the phenotypic responses of 11 *Arabidopsis thaliana* genotypes to different timings (early/mid/late) and frequencies (low/high) of temperature stress on plants which were not sampled for leaves for use in follow-up experiments (see main text). Shown are *F*-ratios and *P*-values, the latter highlighted in bold when below 0.05. Novel significant results compared to the original model are underlined.

|  |  | Flowering time | | Plant architecture | | Aboveground biomass | | Reproductive allocation | | Fecundity | |
| --- | --- | --- | --- | --- | --- | --- | --- | --- | --- | --- | --- |
|  | d.f. | *F*-ratio | *P*-value | *F*-ratio | *P*-value | *F*-ratio | *P*-value | *F*-ratio | *P*-value | *F*-ratio | *P*-value |
| Stress Timing (T) | 2 | 0.15 | 0.859 | 14.36 | **< 0.001** | 3.06 | **0.048** | 7.12 | **< 0.001** | 3.69 | **0.026** |
| Stress Frequency (F) | 1 | 2.05 | 0.154 | 0.08 | 0.776 | 1.80 | 0.181 | 2.31 | 0.129 | 0.87 | 0.352 |
| T × F | 2 | 1.35 | 0.260 | 0.08 | 0.923 | 0.53 | 0.590 | 6.30 | **0.002** | 6.41 | **0.002** |
| Genotype (G) | 10 | 225.40 | **< 0.001** | 28.56 | **< 0.001** | 17.51 | **< 0.001** | 230.33 | **< 0.001** | 85.36 | **< 0.001** |
| G × T | 20 | 7.75 | **< 0.001** | 4.34 | **< 0.001** | 5.89 | **< 0.001** | 4.46 | **< 0.001** | 3.16 | **< 0.001** |
| G × F | 10 | 2.18 | **0.019** | 1.79 | 0.062 | 0.51 | 0.884 | 1.26 | 0.254 | 0.64 | 0.779 |
| G × T × F | 20 | 1.22 | 0.240 | 1.32 | 0.165 | 1.36 | 0.142 | 2.06 | **0.006** | 1.06 | 0.392 |
| Residuals | 279-284 |  |  |  |  |  |  |  |  |  |  |

**Table S3.** Results of linear models testing the phenotypic responses of 11 *Arabidopsis thaliana* genotypes to different timings (early/mid/late) and frequencies (low/high) of temperature stress including flowering time as a covariate. Shown are *F*-ratios and *P*-values, the latter highlighted in bold when below 0.05.

|  |  | Plant architecture | | Aboveground biomass | | Reproductive allocation | | Fecundity | |
| --- | --- | --- | --- | --- | --- | --- | --- | --- | --- |
|  | d.f. | *F*-ratio | *P*-value | *F*-ratio | *P*-value | *F*-ratio | *P*-value | *F*-ratio | *P*-value |
| Leaf sampling | 1 | 11.15 | **0.001** | 68.92 | **< 0.001** | 1.09 | 0.296 | 0.94 | 0.333 |
| Flowering time | 1 | 305.37 | **< 0.001** | 77.83 | **< 0.001** | 2350.00 | **<0.001** | 889.29 | **<0.001** |
| Stress Timing (T) | 2 | 15.68 | **< 0.001** | 1.52 | 0.221 | 33.39 | **< 0.001** | 6.19 | **0.002** |
| Stress Frequency (F) | 1 | 0.03 | 0.854 | 1.80 | 0.180 | 1.76 | 0.186 | 2.02 | 0.156 |
| T × F | 2 | 0.52 | 0.596 | 0.67 | 0.513 | 6.24 | **0.002** | 5.77 | **0.003** |
| Genotype (G) | 10 | 22.29 | **< 0.001** | 20.12 | **< 0.001** | 89.15 | **< 0.001** | 48.22 | **< 0.001** |
| G × T | 20 | 6.64 | **< 0.001** | 5.59 | **< 0.001** | 3.83 | **< 0.001** | 5.14 | **< 0.001** |
| G × F | 10 | 1.45 | 0.155 | 0.53 | 0.866 | 0.31 | 0.977 | 0.65 | 0.767 |
| G × T × F | 20 | 1.18 | 0.263 | 1.48 | 0.084 | 2.10 | **0.004** | 1.02 | 0.436 |
| Residuals | 443-446 |  |  |  |  |  |  |  |  |

**Table S4.** Results of linear models testing the phenotypic responses of 11 *Arabidopsis thaliana* genotypes to different timings (early/mid/late) and frequencies (low/high) of temperature stress including latitude as a random effect. Shown are *F*-ratios and *P*-values, the latter highlighted in bold when below 0.05.

|  |  | Number of fruits | | Aboveground biomass | | Reproductive allocation | | Flowering time | | Plant architecture | |
| --- | --- | --- | --- | --- | --- | --- | --- | --- | --- | --- | --- |
|  | d.f. | *F*-ratio | *P*-value | *F*-ratio | *P*-value | *F*-ratio | *P*-value | *F*-ratio | *P*-value | *F*-ratio | *P*-value |
| Leaf sampling | 1 | 1.47 | 0.226 | 69.05 | **< 0.001** | 2.27 | 0.133 | 0.39 | 0.533 | 9.69 | **0.002** |
| Latitude | 1 | 10.81 | **0.001** | 105.82 | **< 0.001** | 17.39 | **< 0.001** | 4.89 | **0.028** | 124.25 | **< 0.001** |
| Stress Timing (T) | 2 | 3.18 | **0.043** | 2.44 | 0.088 | 14.79 | **< 0.001** | 0.23 | 0.792 | 19.87 | **< 0.001** |
| Stress Frequency (F) | 1 | 0.68 | 0.411 | 1.02 | 0.313 | 0.84 | 0.361 | 1.43 | 0.232 | 0.01 | 0.918 |
| T × F | 2 | 5.66 | **0.004** | 0.85 | 0.428 | 6.74 | **0.001** | 1.67 | 0.189 | 0.37 | 0.692 |
| Genotype (G) | 10 | 144.49 | **< 0.001** | 14.53 | **< 0.001** | 329.06 | **< 0.001** | 395.27 | **< 0.001** | 36.50 | **< 0.001** |
| G × T | 20 | 4.99 | **< 0.001** | 6.63 | **< 0.001** | 4.73 | **< 0.001** | 7.97 | **< 0.001** | 6.53 | **< 0.001** |
| G × F | 10 | 0.73 | 0.694 | 0.65 | 0.769 | 0.82 | 0.609 | 1.67 | 0.107 | 1.54 | 0.123 |
| G × T × F | 20 | 0.87 | 0.621 | 1.34 | 0.148 | 2.47 | **< 0.001** | 0.78 | 0.743 | 1.18 | 0.265 |
| Residuals | 447-454 |  |  |  |  |  |  |  |  |  |  |

**Figure S1.** Fecundity of 11 *Arabidopsis thaliana* genotypes under continuous normal conditions (*n* = 8) and continuous stress conditions (*n* = 3). Error bars indicate 1 SE.


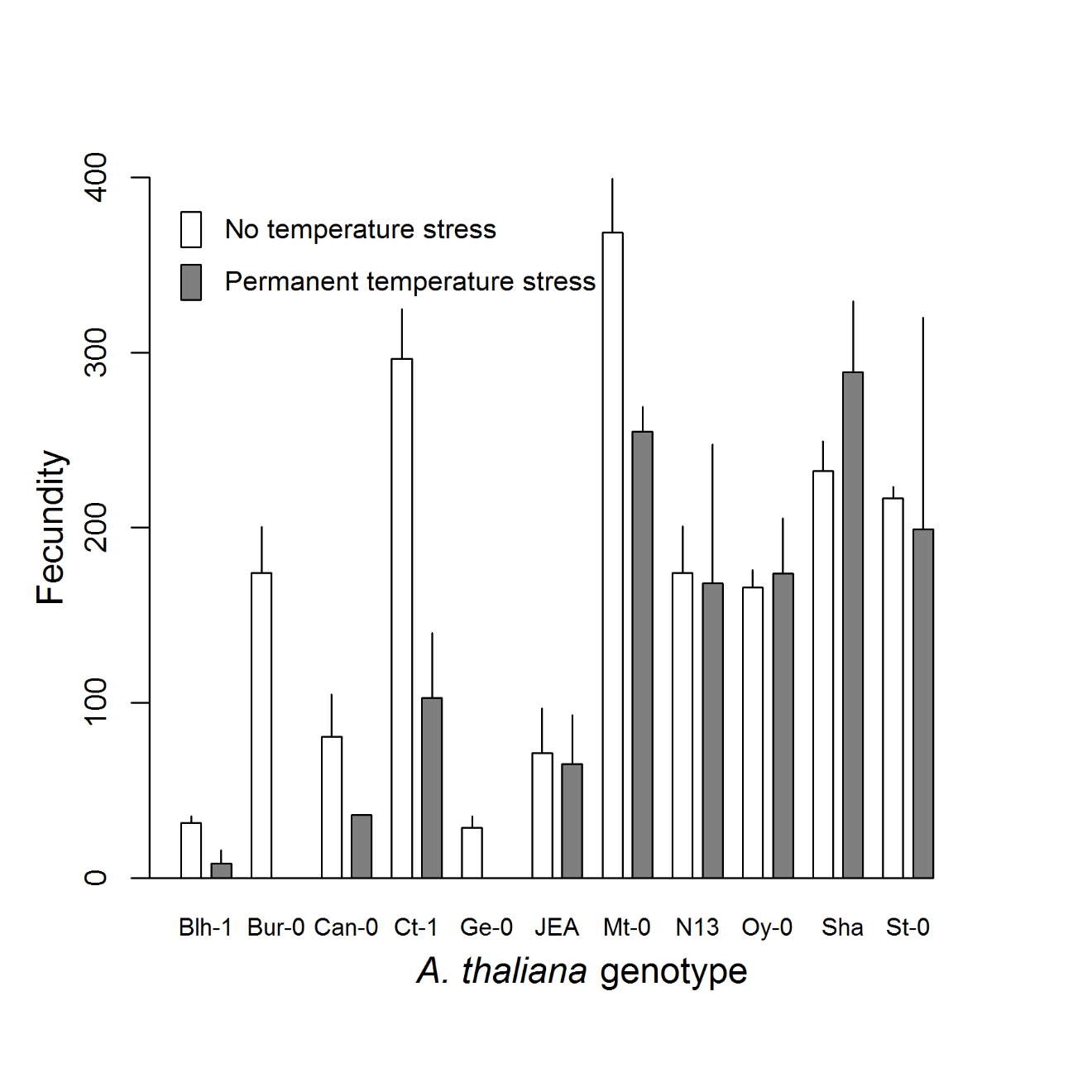


**Figure S2.** Mean responses of (A) reproductive allocation and (B) fecundity to stress timing and frequency across 11 *Arabidopsis thaliana* genotypes.


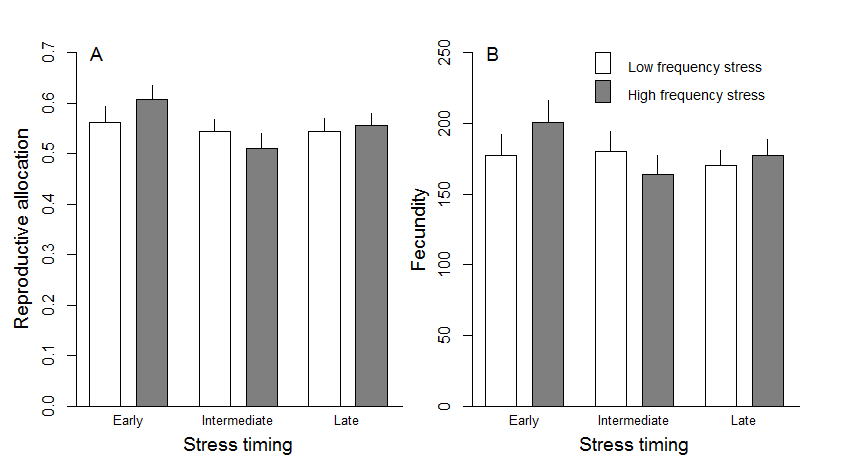


**Figure S3.** Responses of 11 *Arabidopsis thaliana* genotypes to three different timings and two different frequencies of temperature stress in reproductive allocation. Solid lines indicate responses under low stress frequency and dashed lines under high stress frequency. Genotype names are indicated in the top left corner of the panels.


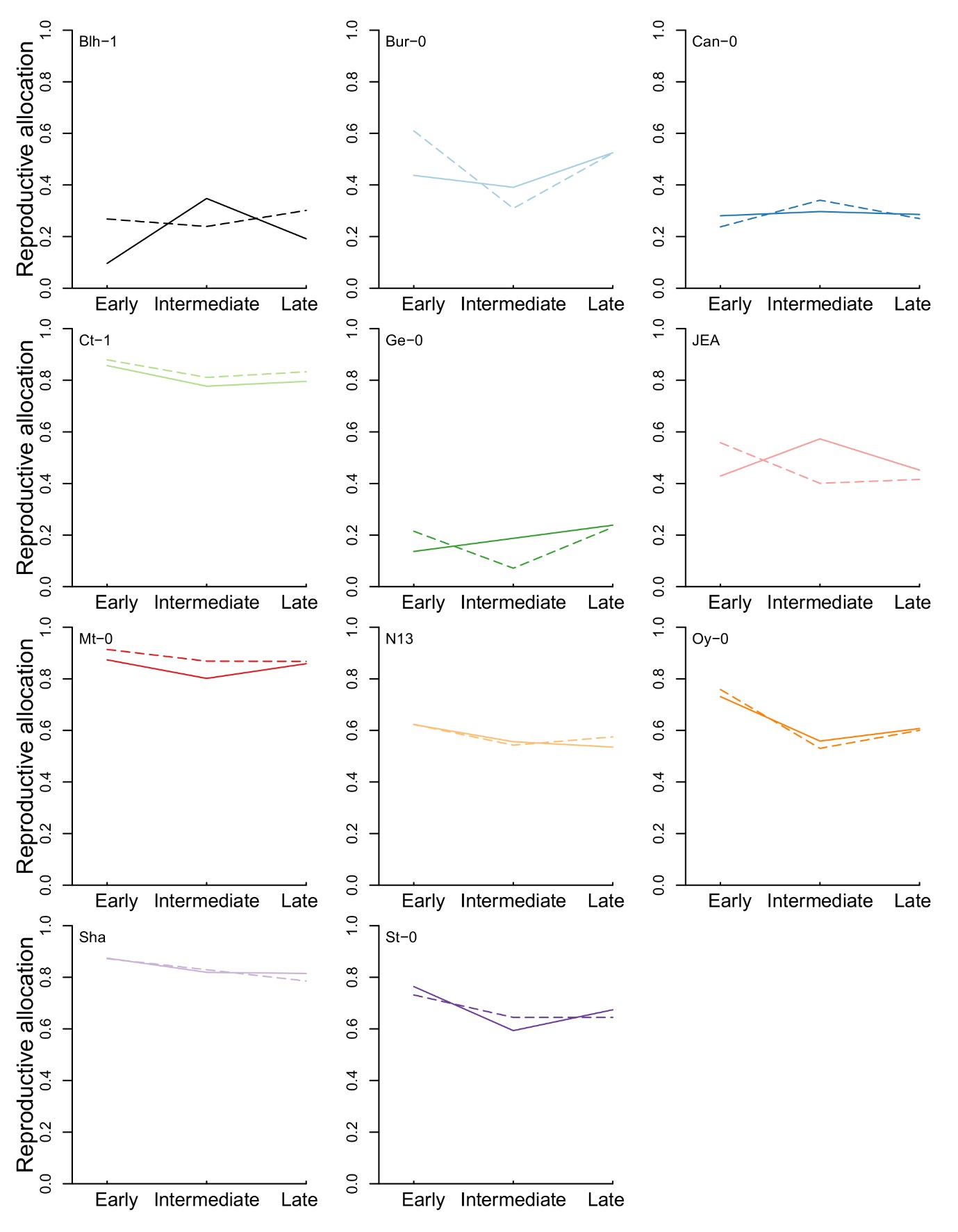

Supplement: Supplement Material [file ply043_suppl_supplement_material.docx]
